# Effector-triggered defence against apoplastic fungal pathogens

**DOI:** 10.1016/j.tplants.2014.04.009

**Published:** 2014-08

**Authors:** Henrik U. Stotz, Georgia K. Mitrousia, Pierre J.G.M. de Wit, Bruce D.L. Fitt

**Affiliations:** 1 School of Life and Medical Sciences, University of Hertfordshire, Hatfield, AL10 9AB, UK; 2 Wageningen University and Research Centre, Laboratory of Phytopathology, Droevendaalsesteeg 1, 6708 PB Wageningen, The Netherlands

**Keywords:** apoplastic fungal pathogens, cell wall, extracellular matrix, receptor-like protein, *R* gene-mediated resistance

## Abstract

*R* gene-mediated host resistance against apoplastic fungal pathogens is not adequately explained by the terms pathogen-associated molecular pattern (PAMP)-triggered immunity (PTI) or effector-triggered immunity (ETI). Therefore, it is proposed that this type of resistance is termed ‘effector-triggered defence’ (ETD). Unlike PTI and ETI, ETD is mediated by *R* genes encoding cell surface-localised receptor-like proteins (RLPs) that engage the receptor-like kinase SOBIR1. In contrast to this extracellular recognition, ETI is initiated by intracellular detection of pathogen effectors. ETI is usually associated with fast, hypersensitive host cell death, whereas ETD often triggers host cell death only after an elapsed period of endophytic pathogen growth. In this opinion, we focus on ETD responses against foliar fungal pathogens of crops.

## Highlights

• ETD is triggered by RLPs that engage the receptor-like kinase SOBIR1.
• ETD triggers cell wall-related defence responses.
• ETD does not eliminate apoplastic pathogens.

## Resistance against apoplastic leaf pathogens of crops

**Figure 1:**
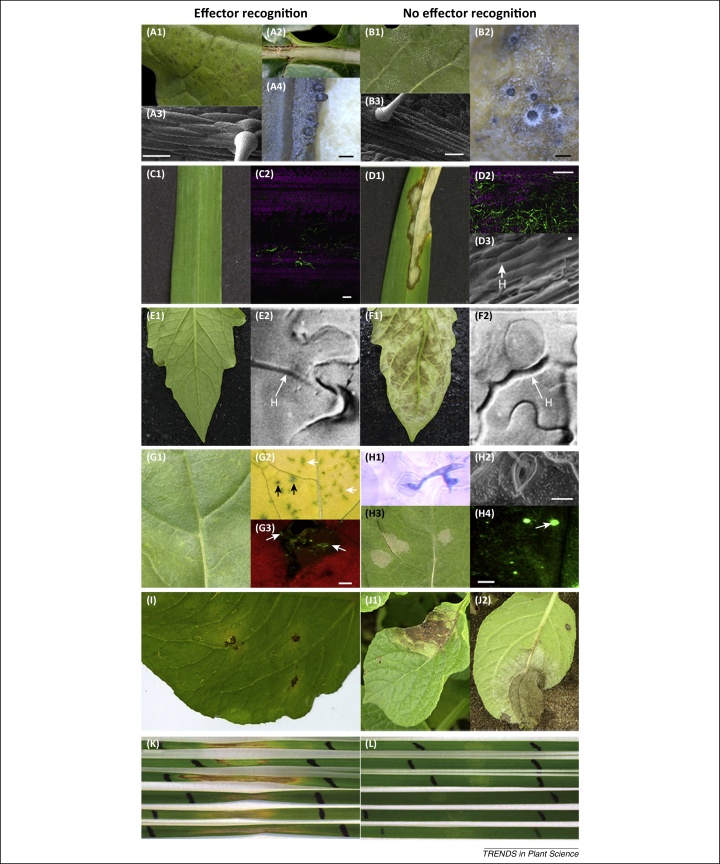
Phenotypes of effector-triggered defence (ETD), effector-triggered immunity (ETI), or effector-triggered susceptibility (ETS) associated with recognition of effectors from representative fungal or oomycete leaf pathogens (featured in Table 1 or Table 2, main text) by contrast with phenotypes associated with nonrecognition of these effectors. ETD **(A–H)** involves limited or no macroscopic symptom development when apoplastic fungal leaf pathogen effectors are recognised by the corresponding *R* genes in the individual hosts (A1, A2, C1, E1, G1). The operation of the *R* gene against apoplastic fungal leaf pathogens limits pathogen growth but does not eliminate the pathogen, which can often subsequently sporulate. ETD in the resistant oilseed rape cultivar ‘Imola’ limited asexual sporulation (acervuli) of *Pyrenopeziza brassicae* (light leaf spot) and dark flecking occurred on (A1) the lamina and (A2) especially along the leaf midrib, as observed 23 days post inoculation (dpi) [8]. (A3) The operation of the *R* gene against *P. brassicae* limited subcuticular hyphal growth, as observed 13 dpi in scanning electron micrographs (SEM, scale bar = 100 μm) of leaf surfaces, but (A4) it did not prevent sexual sporulation because *P. brassicae* apothecia subsequently developed on senescent leaves (scale bar = 0.5 mm). (B3) By contrast, on a susceptible oilseed rape cultivar, extensive subcuticular hyphal growth was observed at 13 dpi (SEM, scale bar = 100 μm), (B1) followed by asexual sporulation (acervuli); (B2) apothecia subsequently developed on senescent leaves (scale bar = 0.5 mm). (C1) Recognition of the *Rhynchosporium commune* (leaf blotch) NIP1 effector by the corresponding Rrs1 receptor of the resistant barley cultivar Turk was not associated with macroscopically visible symptom development, whereas (D1) necrotic lesions developed by 21 dpi with a *Δnip1 R. commune* isolate [11]. (C2) Limited colonisation and asexual sporulation were observed 21 dpi on the resistant barley cultivar Atlas 46 inoculated with the *R. commune* transformant T-R214-GFP (confocal imaging) in contrast to (D3) extensive sub-cuticular hyphal (H) growth of *R. commune* observed by 17 dpi on susceptible barley leaves (SEM, scale bars 10 μm) and (D2) extensive colonisation and sporulation on the susceptible cultivar Atlas by 21 dpi. (E1) ETD operated in a resistant tomato inoculated with *Cladosporium fulvum* (leaf mould) that did not develop any visible symptoms by 14 dpi. (F1) By contrast, the pathogen grew extensively on a susceptible tomato cultivar, with mould developing as light brown patches in which conidiophores erupted through the stomata to produce asexual spores. (E2) ETD against *C. fulvum* growing in the apoplast of a tomato was associated with cell-wall enforcement (black arrow) without visible cell death early after inoculation (3 dpi) but (F2) no cell-wall enforcement had taken place on susceptible tomato plants at 3 dpi with the virulent *C. fulvum* race (H: pathogen hyphae, white arrow) [75]. (G1) ETD triggered by the *Leptosphaeria maculans* (phoma leaf spot) AvrLm6 effector when it was recognised by the Rlm6 receptor on the resistant oilseed rape cultivar DarmorMX did not involve symptom development by 11 dpi with ascospores (without wounding) [32]. (G2) Small dark spots (black arrows) and green islands (white arrows) were observed on DarmorMX 18 dpi when the leaf started to senesce. (G3) There was a necrotic response on leaves of DarmorMX associated with dead plant cells (lack of red chlorophyll fluorescence); however, the pathogen was alive within these small necrotic areas (white arrows) after inoculation with conidia of GFP-expressing *L. maculans*, when viewed under a fluorescent microscope (inoculation with wounding) (scale bar 200 μm). When there was no effector recognition (H1, at 22 h post inoculation) (H2, 42 h post inoculation (SEM, scale bar 10 μm)), germ tubes produced from *L. maculans* ascospores penetrated stomata on oilseed rape leaves [76]. (H3) There was extensive cell death and lesion formation (grey, >2 mm in diameter) on leaves of Darmor (without *Rlm6*) 11 dpi with ascospores of *L. maculans* carrying the effector gene *AvrLm6*. (H4) When there was no recognition of the AvrLm6 effector, the pathogen produced an extensive hyphal network with pycnidia, as demonstrated by using a GFP-expressing *L. maculans* isolate carrying the effector gene *AvrLm6* (white arrows) (scale bar 200 μm) before growing along the leaf petiole to the stem, the organ in which sexual sporulation occurs. In contrast to ETD, ETI **(I–J)** resulted in a macroscopic hypersensitive response on resistant potato (genotype 7644-17, derived from *Solanum avilesii* genotype 478-2) when production of the Rpi-avl1 protein operated against the corresponding *Phytophthora infestans* (late blight) effector. When there was no recognition of pathogen effectors (J1), typical late blight lesions with necrosis and chlorosis developed after 13 dpi with *P. infestans* isolate IPO-C on the susceptible cultivar ‘Nicola’ in a field experiment with (J2) *Phytophthora infestans* sporulating in chlorotic areas on the lower surface of the leaf. In contrast to ETD, ETS (K) results in programmed cell death (PCD) and the pathogen proliferated by 5 days post infiltration with isoforms of the host-selective toxin ToxA from *Phaeosphaeria nodorum* (glume blotch) in the wheat line BG261 that carries the sensitivity gene *Tsn1*. (L) No obvious necrosis was induced in the recessive *tsn1* line BR34 by 5 days post infiltration with the same ToxA isoform [77]. Modified, with permission, from [8] (A2, A3, A4, B1, B2, B3), [11] (C2, D2), [75] (E2, F2), [32] (G1, G2, G3, H3, H4), [76] (H1, H2), and [77] (K,L). C1, D1 provided by Wolfgang Knogge (Leibniz Institute of Plant Biochemistry, Germany); D3 by Kevin King and Jean Devonshire (Rothamsted Research, UK); and I, J1, and J2 by Vivianne Vleeshouwers (Wageningen University, The Netherlands).

Breeding agricultural crops for resistance against pathogens is essential to secure global food production. Despite efforts to control crop diseases, pathogens are estimated to account for losses of 15% of global food production. It is suggested that losses would be almost twice as much without disease control measures, such as crop resistance breeding [1]. There are now opportunities to improve the effectiveness of breeding crops for resistance against damaging pathogens by exploiting new molecular and genetic insights to improve understanding of the defence system of crop plants against pathogens. In this opinion, we focus on the resistance of crops against foliar fungal pathogens that exploit the host apoplast for retrieval of nutrients. Some of these pathogens are globally widespread and of considerable economic importance. They include pathogens that penetrate the host leaf cuticle and then exploit a niche underneath it (e.g., *Pyrenopeziza brassicae*, oilseed rape light leaf spot; *Venturia inaequalis*, apple scab; and *Rhynchosporium commune*, barley leaf blotch, global losses of approximately US$3.5 billion per year). Others enter leaves through stomata, then grow between host mesophyll cells (e.g., *Cladosporium fulvum*, tomato leaf mould; *Leptosphaeria maculans*, oilseed rape phoma stem canker, global losses of approximately US$1 billion per year; and *Zymoseptoria tritici*, wheat septoria leaf blotch, with a global loss of approximately US$5 billion per year) (Table 1, Figure 1). These apoplastic pathogens are all ascomycetes and many of them are dothideomycetes [2].

## Definition of apoplastic ETD

The plant immune system was originally defined as including PTI and ETI [3]. After arrival of a potential pathogen, PTI is rapidly activated by extracellular host recognition of PAMPs by pattern recognition receptors (PRRs); most PRRs are receptor-like kinases with an extracellular receptor domain, a transmembrane domain, and typically a cytoplasmic kinase domain. Pathogens produce effectors to suppress PTI and cause effector-triggered susceptibility (ETS). In resistant hosts, ETI is initiated by intracellular host recognition of pathogen effectors [4,5]. The term ‘PAMPs’ was originally used to describe invariant molecules present in many microbial pathogens that are essential for their survival [6]. PAMPs that fit this definition include flagellin and chitin, which are present in bacteria and fungi, nematodes or insects, respectively. It was proposed to reduce the stringency of this original definition by considering conserved effectors as not significantly different from PAMPs, without knowledge of their essential functions [7]. PTI has sometimes been defined as PRR-triggered immunity [5]. In addition to their original role in PAMP recognition [6], it was proposed that PRRs directly or indirectly recognise effectors produced by apoplastic pathogens [5]. However, it is not universally accepted that resistance against apoplastic pathogens should be considered as PTI [7]; some consider it as ETI [3]. Furthermore, this new definition of PTI does not account for the fact that some *R* genes encode proteins that are similar to PRRs but interact with extracellular effectors [7]. To explain interactions between apoplastic fungal pathogens and their host plants and reconcile these opposing views, it is proposed that this resistance is referred to as ETD.

Given that this resistance does not generally eliminate these apoplastic pathogens (in the cases of *P. brassicae* and *Z. tritici*, the pathogen is subsequently able to complete its sexual life cycle on the resistant host [8]), ‘defence’ is a more appropriate term than ‘immunity’, which is generally associated with death of the pathogen (Table 2). The response is effector triggered and mediated by *R* gene-encoded proteins, thus justifying ETD as a relevant term. Whereas PTI operates against both apoplastic and cell-penetrating filamentous pathogens (referred to as ‘haustorial pathogens’), the mode of *R* gene-mediated resistance against them differs between the two types of pathogen (Box 1).

**Figure 2:**
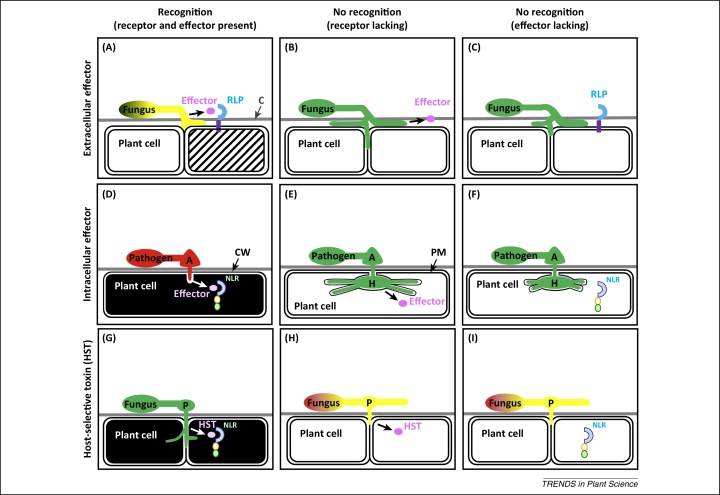
Three types of host response to filamentous leaf pathogens, based on examples from Table 1 or Table 2 (main text). This diagram illustrates specific interactions between single pathogen effectors and corresponding host gene products. In reality, pathogens secrete numerous effectors that directly or indirectly interact with corresponding host gene products. **(A)** Resistance (*R*) gene-mediated effector-triggered defence (ETD) results in incompatible interactions with hemibiotrophic apoplastic fungal leaf pathogens. Extracellular recognition of effectors from fungal pathogens growing underneath the host cuticle (C) (*Rhychosporium commune*, *Pyrenopeziza brassicae*, and *Venturia inaequalis*) or between mesophyll cells (*Cladosporium fulvum*, *Leptosphaeria maculans*, and *Zymoseptoria tritici*) by receptor-like proteins (RLPs) can result in resistance without macroscopically visible host cell death (*C. fulvum* and *R. commune*) (▨). Host cell death typically occurs in only a few cells several days (*C. fulvum* and *L. maculans*) or weeks (*R. commune* and *P. brassicae*) after infection. The pathogen does not die () but can resume growth after host senescence begins or after the immune response is otherwise compromised. **(B)** In compatible interactions, in the absence of an RLP, the host stays alive (**□**) and the virulence function of the effector can promote extensive fungal proliferation (). **(C)** In the absence of the effector, the pathogen may proliferate less (). **(D)***R* gene-mediated effector-triggered immunity (ETI) results in incompatible interactions with obligate biotrophic fungal (*Blumeria graminis* and *Puccinia striiformis*), oomycete (*Bremia lactucae*) pathogens, or some hemibiotrophic oomycete (*Phythophthora infestans*) or fungal (*Magnaporthe grisea*) pathogens. Upon formation of an appressorium (A) to breach the cell wall (CW) and penetrate an epidermal cell, specific fungal or oomycete effectors are secreted and delivered into the host cytoplasm, where recognition by corresponding nucleotide-binding site (NBS) leucine-rich repeat (LRR) receptors (NLRs) occurs. This recognition event triggers a rapid hypersensitive response (typically <1 day after infection) that boosts host defence and usually results in host (■) and pathogen cell death (). **(E)** Compatible interactions lead to the formation of haustoria (H) or a biotrophic interfacial complex through plasma membrane (PM) invaginations. In this case, the host cells stay alive (**□**). The effector stimulates pathogen proliferation (). **(F)** In the absence of the effector that compromises basal plant defence responses, pathogen growth () is slower. **(G)** Effector-triggered susceptibility (ETS) results in compatible interactions with necrotrophic fungal pathogens that secrete host-selective toxins (HSTs). Before entry through the leaf epidermis by means of penetration structures (P) such as hyphopodia (*Phaeosphaeria nodorum*) or appressoria (*Cochliobolus victoriae*), HSTs are released to target specific host proteins that are sensitive to the toxin (some are *R* gene products) and trigger host cell death (■) (typically within a day). Arrows indicate the final cellular destination of effectors of HSTs. Effectors are not injected into but taken up by the host cell. This leads to fungal proliferation (). Membrane invaginations do not occur. Entry into the leaf is also possible through stomata without development of penetration structures (*P. nodorum*). **(H)** In incompatible interactions and absence of host cell death (**□**), the fungal pathogen attempts to penetrate but cannot invade leaves. The pathogen can grow and survive on the plant surface for several days before it dies when nutrients are exhausted (). **(I)** Presence or absence of HST or its target has no impact on superficial growth. Colour codes for molecules and domains, which are not drawn to scale: effector or HST ; LRR domains ; NBS ; coiled-coil or Toll/interleukin-1 receptor domains ; transmembrane domain . NLRs are colour-coded the same for ETI and ETS because the same receptor may confer resistance against a biotrophic pathogen and susceptibility to a necrotrophic pathogen.

In contrast to ETI against haustorial pathogens, ETD responses against apoplastic pathogens are relatively slow and not associated with a fast, orchestrated hypersensitive host cell death response (HR) [3,8–11] (Figure 2A–F). Effectors of apoplastic pathogens are recognised at the cell surface, whereas those of haustorial pathogens are recognised within the cell cytoplasm. The receptors that initiate ETI or ETD differ not only in their domain structures but also in their interactions with other proteins and signalling partners. *R* gene-mediated ETD has some similarities to PTI, in that both are initiated at the cell surface (Box 1).

ETD operates against apoplastic pathogens that are adapted to colonise the intercellular matrix, relying on effectors secreted into the apoplast [12] and not forming haustoria (Table 1, Figure 1E1–F2). Conversely, ETI works against obligate biotrophic filamentous pathogens that produce haustoria (Figure 2E,F) to retrieve nutrients from living plant cells (e.g., *Blumeria graminis*, barley powdery mildew; *Bremia lacucae*, lettuce downy mildew; and *Puccinia striiformis*, wheat yellow rust), and some hemibiotrophic oomycetes (e.g., *Phytophthora infestans*, potato late blight; Figure 1I–J2) and fungi (e.g., *Magnaporthe grisea*, rice blast) [13,14].

## Compromised host defence during initial endophytic growth of apoplastic pathogens

ETD has been studied with plant hosts of several apoplastic foliar fungal pathogens. Those pathogens listed in Table 1 are all ascomycetes and, except for *P. brassicae* and *R. commune*, they are dothideomycetes. Although some dothideomycetes are saprophytes, many are epiphytes, endophytes, or pathogens of plants [2]. Apoplastic pathogens, such as *C. fulvum*, *L. maculans*, *Z. tritici*, *P. brassicae*, *V. inaequalis*, and *R. commune*, do not trigger an effective host defence response during the early stages of invasion (Table 1, Figure 2A–C) but slowly colonise the apoplast to adapt to the constitutive antimicrobial compounds present. For example, *Z. tritici* grows seemingly undetected inside its wheat (*Triticum aestivum*) host for 10–13 days after inoculation [15,16].

Apoplastic pathogens overcome constitutive and induced basal plant defences (PTI) using both offensive and defensive strategies. Among the offensive strategies are: (i) direct penetration of cuticles, using cutinases, by subcuticular pathogens such as *P. brassicae*; and (ii) entry into leaves through stomatal pores by intercellular pathogens such as *L. maculans* (Figure 1H1–2). Furthermore, they also constitutively produce enzymes such as tomatinase and proteases [17,18]. For example, the antifungal activity of the tomato (*Solanum lycopersicum*) glycoalkaloid α-tomatine in its apoplast is overcome by *C. fulvum* detoxifying it into the less fungitoxic compounds tomatidine and oligosaccharide lycotetraose [17]. The lycotetraose released might even serve as a carbon source for the pathogen. A *C. fulvum* strain deficient in GH10 tomatinase was less virulent, particularly during later stages of colonisation around 10 days post-inoculation (dpi). At this later stage, the biomass of wild type *C. fulvum* increased dramatically [17]. Another offensive mechanism is the destruction by cleavage of plant constitutive or induced class IV chitinases that can hydrolyse fungal cell walls [18].

Among the defensive strategies, protection against constitutive apoplastic tomato chitinases is achieved by *C. fulvum* secreting into the apoplast the Avr4 effector that binds to chitin in its cell walls to protect them from hydrolysis [19]. Homologues of *Avr4* occur in several dothideomycetes, making *Avr4* a common effector gene [12]. It is conceivable that protection of fungal cell walls by Avr4 against chitinases is incomplete and that some chito-oligosaccharides are still released. Chito-oligosaccharide-triggered basal plant defence is avoided by some apoplastic pathogens because they produce chitin-binding effectors that sequester these oligosaccharides. *Cladosporium fulvum* produces a LysM domain-containing effector protein, Ecp6, that sequesters chito-oligosaccharides, preventing them from inducing chitin-triggered immunity [20]. *Zymoseptoria tritici* also secretes different types of LysM effector during the endophytic phase of plant colonisation by the pathogen, either to protect itself against plant chitinases or to sequester chitin-oligosaccharides to prevent PTI [21]. LysM effectors have also been found in other types of pathogen, including *M. grisea* [22]. *Ecp6* occurs even more widely than *Avr4*. Thus, effectors of apoplastic fungi target basal defence components occurring in the apoplast.

The numerous proteases expressed by *Z. tritici* during the endophytic phase may compromise host defence enzymes, such as chitinases and β-glucanases, and amino acids released might serve as pathogen nutrients [18,23]. Basal wheat defence is avoided by *Z. tritici* through production of a specific set of adapted plant cell wall-degrading enzymes (CWDEs) [16,24]. Host-adapted CWDEs are produced during the early endophytic phase of colonisation [16]. During the subsequent necrotrophic growth phase, *Z. tritici* produces a different set of CWDEs to permit extensive colonisation of the host. However, these CWDEs are initially expressed at low concentrations during the endophytic growth phase to avoid recognition by the plant immune system [16].

By contrast, basal host defences against cell-penetrating, haustoria-forming pathogens are suppressed by effectors that are active in the host cytoplasm; these effectors can induce an intracellular ETI resistance response [3–5] (Figure 2D–F). Furthermore, a similar combination of strategies, but with entry associated with hyphopodia or penetration of stomatal pores, is used by host-specific necrotrophic fungal pathogens that deliver host-selective toxins (HSTs) inside plant cells to induce ETS [25] (Figure 2G–I).

## Timing of host recognition of apoplastic pathogen effectors

In contrast to the fast timing of ETI in the host cytoplasm (Figure 2D–F), or ETS (Figure 2G–H; e.g., *Phaeosphaeria nodorum*, Figure 1K,L), triggered by haustoria or biotrophic interfacial complex (BIC)-forming biotrophic hemibiotrophic, or necrotrophic filamentous pathogens, respectively (Table 2), triggering of extracellular ETD (Figure 2A–C) by apoplastic fungal pathogens is generally slow. This is because the maximum expression of their effector genes occurs only after an initial phase of endophytic growth and the effectors often need to be processed to exert their virulence function or to be recognised by RLPs (Table 1). Although expression of the *Avr4* effector gene has been detected as early as 1 day after stomatal entry by *C. fulvum* [26], maximum expression occurs at 4–6 dpi; a similar pattern is observed for *Avr9* [27]. Both Avr4 and Avr9 effectors of *C. fulvum* contain a functional glycosylation site (NSS for Avr9 and NLS for Avr4). Glycosylation of both effectors may affect their ability to induce ETD and their recognition by RLPs [28].

Similarly, ETD defence responses by oilseed rape (*Brassica napus*) against *L. maculans* colonisation are slow because expression of the *AvrLm1*, *AvrLm6*, *AvrLm4-7*, and *AvrLm11* effectors of *L. maculans* (Figure 1G1–H4) is limited at 3 dpi, reaching a maximum at 7 dpi [29–31]. Few pathogen hyphae are present at 3 dpi but by 7 dpi the mycelial mass has increased [30]. A microscopic reaction involving cell death occurs 8 days after leaf inoculation of *B. napus* cv. DarmorMX (carrying the resistance gene *Rlm6*) with ascospores of a *L. maculans* isolate carrying the corresponding *AvrLm6* gene [32]. However, there has been subcellular evidence of necrosis occurring by 6 days after infiltration of oilseed rape cotyledons with *L. maculans* conidia [33]. An *AvrLm1*-dependent increase in expression of the pathogenesis-related (PR) gene *PR1* was detected at 5 days after infiltration with conidia, at the same time as an increase in salicylic acid production [33]. These host defence responses against *L. maculans* do not eliminate the pathogen, which can be re-isolated from resistant host tissue and cultured [32].

ETD against *Z. tritici* is also expressed only after the pathogen has entered wheat leaves through stomata and grown endophytically between mesophyll cells in leaves for the first week after inoculation [34]. ETD operates in the resistant host at approximately 10 dpi when the rapid switch from endophytic to necrotrophic growth occurs in the susceptible host [15,16], but this ETD is not associated with host cell death [35].

Plant defence against apoplastic pathogens that occupy a subcuticular niche is also generally slower than defence against haustoria or BIC-forming filamentous pathogens. Although the effect of *R* gene-mediated resistance could be detected as early as 3 dpi of barley (*Hordeum vulgare*) leaves with *R. commune* (Figure 1C1–D3), cell death only occurred at 21 dpi [11]. In the presence of the resistance gene *Rrs1*, both subcuticular growth and sporulation of an avirulent isolate were impeded [11,36]. Expression of PR genes *PR1*, *PR5*, *PR9*, and *PR10*, all of which encode extracellular proteins, is induced as early as 1 dpi in the *Rrs1*-containing genotype that is responsive to the corresponding effector NIP1 [37]. In the susceptible host, induction of these *PR* genes occurs 2 days later [37].

Expression of oilseed rape resistance against *P. brassicae* (Figure 1A1–B3) is also slow, operating from 13 to 36 dpi to prevent a 300-fold increase in pathogen biomass [8]. The operation of resistance, associated with the *PBR2* gene, triggers a collapse of epidermal cells or ‘necrotic flecking’ accompanied by little or no asexual sporulation. However, this resistance does not interfere with subsequent sexual sporulation on senescent leaves 36 dpi [8].

These slow ETD responses are in contrast to ETI induced by haustoria or BIC-forming filamentous pathogens. Expression of effector genes in these pathogens evokes a fast HR, generally resulting in death of the host cells and the pathogen. Effector genes of *P. infestans*, for instance, reach a maximum expression 2 dpi of potato (*Solanum tuberosum*) [38]. HSTs, which are effectors of host-specific necrotrophic fungal pathogens, are even expressed constitutively (Table 2). Effector recognition is equally fast, triggering host cell death within 1–2 dpi of wheat by *P. nodorum* or oats (*Avena sativa*) by *Cochliobolus victoriae* (Table 2) [25,39–41].

## Recognition of apoplastic pathogen effectors is mediated by RLPs

ETD involves host recognition of apoplastic effectors in intercellular spaces by cell surface RLPs that are integral plant membrane proteins containing an extracellular leucine-rich repeat (eLRR) domain and a short cytoplasmic tail without a signalling motif [12,42–44] (Figure 2A). Recognition of apoplastic effectors and triggering of RLP-mediated defence is described best in the tomato–*C. fulvum* pathosystem (Table 1). Avr2 is a cysteine protease inhibitor that binds to and inhibits the plant protease Rcr3 [45]. The corresponding RLP, Cf-2, acts as a guard to survey the modulation of Rcr3 [46]. Cf-2 probably recognises the Avr2-modulated Rcr3 protein because Avr2 mutants with reduced Rcr3 binding are impaired in their ability to trigger Cf-2-mediated host cell death [47]. Three RLPs, Cf-2, Cf-4, and Cf-9, were all shown to interact with the receptor-like kinase (RLK) SOBIR1/EVR for downstream signalling and defence [48]. It is suggested that SOBIR1 functions specifically in receptor complexes with RLPs involved in RLP-mediated ETD [49] and RLP-mediated plant development (CLV2); SOBIR1 does not engage with BAK1 to trigger PTI (Box 1). However, it should be noted that complexing of SOBIR1 with RLPs has usually been studied in the absence of their corresponding ligands and it is expected that ligand-triggered RLP-mediated defence or development responses require new complex associations or dissociations to amplify defence or development signalling. It has also been suggested that SOBIR1 is involved in stabilisation and trafficking of RLPs [48,49].

RLPs are also involved in oilseed rape resistance against *L. maculans* and apple (*Malus domestica*) resistance against *V. inaequalis* [43,50,51] (Table 1). *AvrLm1* encodes a secreted mature protein of 21 kDa with a single cysteine residue [52]. *AvrLm1* genetically interacts with *LepR3*, the first *R* gene cloned from *B. napus* to encode an RLP [43,44]. One of four *R* gene paralogues, *HcrVf2*, is necessary and sufficient for apple resistance against *V. inaequalis* strains that carry the cognate effector gene *AvrRvi6* [42,53]. It is also likely that NIP1, an effector of the barley leaf blotch pathogen *R. commune*, is recognised at the cell wall because it binds to a plasma membrane protein and stimulates H^+^-ATPase activity [54]. Resistance to NIP1-producing strains of *R. commune* is governed by the *Rrs1* gene [55]. Although this opinion focuses on apoplastic fungal pathogens of leaves, ETD responses against vascular pathogens, such as *Verticillium dahliae*, are also mediated by RLPs that require SOBIR1, such as Ve1 [48,56].

By contrast, pathogen effectors that are targeted to the host cytoplasm are directly or indirectly recognised by nucleotide binding site (NBS) LRR receptors (NLRs) located in the cytoplasm [57–60] (Figure 2D,G). These recognition events usually trigger a fast HR or programmed cell death [41] that leads to resistance against biotrophic pathogens, such as *Blumeria graminis* on barley, *Bremia lactucae* on lettuce (*Lactuca serriola*), and *Puccinia striiformis* on wheat [61–63], and some hemibiotrophic pathogens, such as *P. infestans* on potato and *Magnaporthe grisea* on rice (*Oryza sativa*) (ETI) [64,65], or susceptibility to some host-specific necrotrophic pathogens (ETS) (Table 2). However, it should be noted that it is still not clear whether the HR is a cause or consequence of resistance, because induction of a HR and resistance can sometimes be separated [66].

## Exploitation for crop breeding for resistance and food security

There are now unprecedented opportunities to exploit new genomic information about crop hosts and pathogens and new knowledge about the operation of host defence against pathogen attack in plant breeding to produce crops with more durable resistance against damaging pathogens [4]. For example, if resistance against all apoplastic pathogens is mediated by genes encoding RLPs, for crops attacked by apoplastic pathogens, it should be possible to screen their genomes, especially those regions identified as containing loci for resistance against these pathogens, specifically for *R* genes that encode RLPs. The genes identified can then be considered as candidate ETD resistance genes. Such methods have already been used to identify ETI resistance genes encoding cytoplasmic NLRs [67]. To identify the most useful RLPs as candidate *R* gene targets for breeding, they could be functionally analysed for responsiveness to corresponding effectors of apoplastic fungal pathogens that are essential for virulence. Breeders could subsequently design molecular markers to combine several functional *R* genes encoding RLPs in elite crop cultivars to increase the durability of the cultivar resistance against apoplastic pathogens.

To preserve valuable sources of resistance and avoid catastrophic epidemics associated with changes in virulence spectra within pathogen populations that render ineffective single *R* genes widely deployed over large areas [68], there is also a need for schemes to guide the deployment of different *R* genes in space and time by farmers selecting which cultivars to grow in their fields. This requires knowledge of the *R* genes deployed in different commercial cultivars and a web-based scheme to guide farmers by grouping cultivars according to the *R* genes they contain. Such schemes are currently being operated in France and Australia for management of *L. maculans* [69]. If effective *R* genes operating against apoplastic pathogens and the virulence spectra of their attacking pathogens can be identified more rapidly and deployed more carefully, they will make a substantial contribution to sustainable crop protection and improved food security.

## Figures and Tables

**Figure I:**
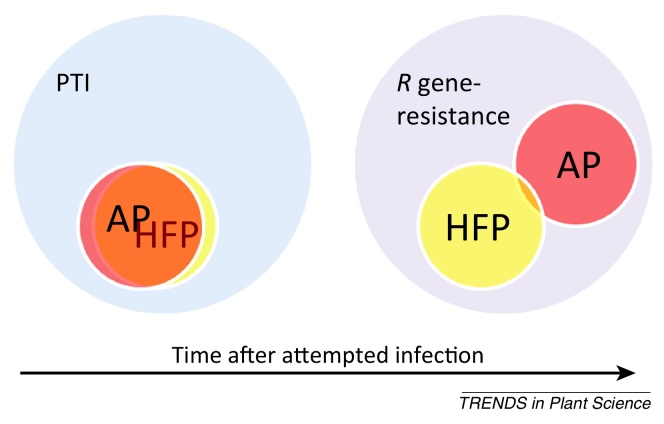
Pathogen-associated molecular pattern (PAMP)-triggered immunity (PTI) is conserved and this host defence response against apoplastic pathogens (AP) and haustoria-forming (and other cell-penetrating) pathogens (HFP) does not generally differ. Although *R* gene-mediated resistance operates against both AP and HFP, these defence responses differ in that effector-triggered immunity (ETI) and effector-triggered defence (ETD) operate against HFP and AP, respectively. Specific differences are explained in Table I.

**Table 1:** Components of the phenotype of RLP-mediated resistance operating in leaves of arable and/or horticultural crops against hemibiotrophic fungal pathogens that colonise an apoplastic niche

Pathogen (hemibiotrophic)^a^	Niche^b^	Host^c^	R gene^d^	Phenotype					Refs
				Host cell death^e^ (dpi)	Pathogen death	Limits pathogen biomass^f^	Limits asexual sporulation^g^	Prevents sexual sporulation^h^	
*Pyrenopeziza brassicae*^i^	Subcuticular	*Brassica napus*	*PBR1*, *PBR2*	CD (<14)	No	Yes	Yes	No	[8]
*Venturia inaequalis*^j^	Subcuticular	*Malus domestica*	***HcrVf2***	CD (2-11)	No	Yes	Yes	n/k	[42,53,70]
*Rhynchosporium commune*^k^	Subcuticular	*Hordeum vulgare*	*Rrs1*	CD (<21)	No	Yes	Yes	n/k	[11,37,55,71]
*Cladosporium fulvum*^l^	Intercellular (mesophyll)	*Solanum lycopersicum*	***Cf-2,4,4E,9***	CD (<4)	No	Yes	Yes	n/k	[72,73]
*Leptosphaeria. maculans*^m^	Intercellular (mesophyll)	*B. napus*	*Rlm6*, ***LepR3***	CD (<7)	No	Yes	Yes	Yes	[32,43]
*Zymoseptoria tritici*^n^	Intercellular (mesophyll)	*Triticum aestivum*	*STB* genes?	No	No	Yes	Yes	No	[74]

a These hemibiotrophic pathogens are in order with most ‘biotrophic’ first and most ‘necrotropic’ last. This ranking may be somewhat subjective but *P. brassicae* is the most ‘biotrophic’ because its pathogenicity cannot be maintained in artificial culture.

b The niche occupied by these hemibiotrophic pathogens after initial infection of leaf tissues at the time when the *R* gene is operating during the endophytic growth phase; frequently, these pathogens later switch to a necrotrophic phase and occupy niches in other plant tissues.

c Host for which the phenotype of *R* gene action was studied; sometimes, the pathogen also attacks closely related hosts.

d Specific *R* gene(s) that has been studied. Cloned RLP genes are in bold; these all encode RLPs. Mapped *R* genes with a described phenotype are underlined. It is assumed that the less well-characterised *R* genes also encode RLPs.

e The mode of host cell death is not well characterised and is simply referred to as cell death (CD).

f Evidence obtained by microscopy, ELISA, or quantitative PCR that the pathogen grows less extensively in host plants with the *R* gene than in those without it.

g Evidence of no or limited asexual sporulation associated with colonisation by the pathogen.

h Evidence of sexual sporulation associated with subsequent colonisation of senescent leaf tissue by the pathogen.

i Occupies a subcuticular niche. It is suggested that there are two resistance loci. One locus (*PBR2*) on chromosome A1 is associated with necrotic flecking and a limitation of asexual sporulation; subsequent sexual sporulation is not affected.

j Spores form germ tubes that penetrate the cuticle and proliferate into subcuticular stromata. Host damage does not occur until onset of asexual sporulation. Speed of the resistance response depends on the *R* gene. *HcrVf2* triggers cell death. The effect of *R* gene-mediated resistance on sexual reproduction is not known (n/k).

k The *Rrs1* gene has not been cloned but it interacts genetically with the *nip1* gene encoding a Cys-rich secreted peptide. Collapse of single or a few epidermal cells was reported to occur in both resistant and susceptible plants but then to continue only in susceptible plants. Asexual conidia are produced on resistant hosts, although less extensively than on susceptible hosts. Given that the teleomorph has not yet been found, it is not possible to assess an effect on sexual sporulation, even though population studies suggest that sexual reproduction and different mating types occur on the same leaf.

l After entry through stomata, hyphae are arrested in the substomatal cavity after contact with mesophyll cells. Mesophyll cells in close proximity to hyphae undergo CD, which is controlled by *Cf* genes. The pathogen does not necessarily die and can sporulate in genotypes carrying *Cf-1* or *Cf-3* genes. Although different mating types exist, the sexual stage has not been identified.

m The pathogen penetrates through stomata and then grows in intercellular spaces. In the resistant host, CD around the site of penetration is associated with containment of the pathogen. In the susceptible host, there is extensive CD, lesion formation, and production of asexual spores in pycnidia, followed by spread of the pathogen along the leaf petiole to the stem, where sexual sporulation occurs. The *R* gene *LepR3* has recently been cloned.

n *STB* genes are not associated with host cell death, but do reduce pathogen biomass and asexual sporulation without preventing sexual sporulation.

**Table 2:** Components of the phenotype of nucleotide binding site (NBS) leucine-rich repeat (LRR) receptor (NLR)-mediated resistance or susceptibility operating in leaves of arable and horticultural crops against obligate biotrophic, hemibiotrophic or necrotrophic fungal or oomycete pathogens that colonise an intracellular niche

Pathogen (obligate biotrophic; hemibiotrophic; or necrotrophic)^a^	Fungus (F) or oomycete (O)	Niche^b^	Host^c^	NLR gene^d^	Phenotype					Refs
					Host cell death^e^ (dpi)	Pathogen death	Limits pathogen biomass^f^	Limits asexual sporulation	Limits sexual sporulation	
*Blumeria graminis* (b)^g^	F	Intracellular (epidermal)	*Hordeum vulgare*	*Mla* genes	HR (<1)	Yes	n/a^h^	n/a	n/a	[61]
*Bremia lactucae* (b)^i^	O	Intracellular (epidermal)	*Lactuca serriola*	*Dm3*	HR (<1)	Yes	n/a	n/a	n/a	[63]
*Puccinia striiformis* (b)^j^	F	Intracellular (mesophyll)	*Triticum aestivum*	*Yr1*	HR (<1)	Yes	n/a	n/a	n/a	[62]
*Phytophthora infestans* (h)^k^	O	Intracellular (epidermal)	*Solanum tuberosum*	*R1*, *R3b*	HR (1-2)	Yes	n/a	n/a	n/a	[65]
*Magnaporthe grisea* (h)^l^	F	Intracellular (epidermal)	*Oryza sativa*	*Pi-ta*	HR (<2)	Yes	n/a	n/a	n/a	[64]
*Phaeosphaeria nodorum* (n)^m^	F	Dying host cell (epidermal)	*Triticum aestivum*	*Tsn1*, *Snn1-4*	PCD (<2)	No	No	No	No	[25]
*Cochliobolus victoriae* (n)^n^	F	Dying host cell (epidermal)	*Avena sativa*	*Vb*	PCD (<1)	No	No	No	n/k	[39–41]

a Pathogens are categorised as obligate biotrophic (b), hemibiotrophic (h), or necrotropic (n).

b The niche occupied by biotrophic, hemibiotrophic or necrotrophic pathogens after initial infection of leaf tissues at the time when the NLR gene is operating; frequently, hemibiotrophic pathogens subsequently switch from a biotrophic to a necrotrophic mode and may then occupy niches in other plant tissues.

c Host for which the phenotype of the NLR gene action was studied; sometimes, the pathogen also attacks other related hosts.

d NLR genes confer resistance against biotrophic and hemibiotrophic pathogens, but they can be hijacked by host-selective toxins from necrotrophic fungi. This list of NLR gene(s) is not exhaustive.

e The mode of host cell death can be clearly defined in the case of the hypersensitive response (HR). DNA laddering and heterochromatin condensation are used as evidence for programmed cell death (PCD).

f The presence of an NLR gene promotes more extensive growth and sporulation of the necrotrophic pathogens that produce the corresponding host-selective toxin.

g Colonisation of epidermal cells by means of haustoria. *Mla* genes are associated with a rapid HR.

h Not applicable (n/a) for obligate biotrophic pathogens because the HR generally causes pathogen death; it is not possible to assess these aspects of the phenotype of resistance.

i Penetration of epidermal cells leads to primary vesicle formation, which is impeded by *Dm* genes and associated with an HR.

j Colonisation of mesophyll cells by means of haustoria. *Yr1* is associated with a rapid HR.

k Occupies epidermal niche after penetration; the HR impairs the pathogen.

l The *Pi-ta* gene triggers an HR that results in death of the pathogen.

m As the Tox1 protein is recognised by Ssn1, the pathogen penetrates the epidermis in the presence of light and causes PCD. *ToxA* is constitutively expressed and the effector is recognised by Tsn1, which contains NBS-LRR domains characteristic of cytosolic NLRs.

n Germinating spores produce victorin before penetration of susceptible oats expressing the *Vb* gene. Although *Vb* has not yet been cloned, *LOV1*, which confers victorin susceptibility in *Arabidopsis thaliana*, contains an NBS-LRR domain. *Vb* may well be identical to *Pc-2*, which confers resistance to the biotrophic rust fungus *Puccinia coronata*. *Cochliobolus victoriae* has mating type genes but to our knowledge the sexual stage has not been discovered. PCD in response to victorin has also recently been referred to as victorin-induced cell death (VICD).

**Table I:** Differences in resistance responses of hosts between those operating against haustoria-forming pathogens (HFPs) and those operating against apoplastic fungal leaf pathogens (APs)

	HFP and AP	HFP	AP
	PTI	ETI	ETD
Speed of resistance response	Rapid (within hours)^a^	Fast (<2 dpi)^b^	Slow (4^c^–36^d^ dpi)
Triggered by^e^	PAMPs	Intracellular effectors	Apoplastic effectors
Mediated by^f^	PRRs	NLRs	RLPs
Effector domain^g^	Kinase	TIR or CC	N/A
Dimerisation^h^	Heterodimers	Homodimers	Heterodimers
Interacting proteins^i^	BAK1	Transcription factors	SOBIR1/BAK1
Cell death^j^	Not typically	Fast HR	Slow CD sometimes

a PTI or nonhost resistance is rapid, occurring within hours of attempted infection [78]. *Cladosporium fulvum* growth is stopped during attempted penetration of stomata of tobacco leaves.

b The speed of ETI is fast; an intracellular response typically occurs 1–2 days after infection [62].

c ETD occurs several days after infection in the tomato mesophyll layer [e.g., *C. fulvum* penetrates stomata 3 days post-inoculation (dpi) and *Avr9* triggers host cell death 4 dpi] [27]. Nevertheless, the pathogen is still alive 7 dpi [26].

d ETD occurs up to 36 days after infection (e.g., barley recognition of the *Rhynchosporium commune* NIP1 effector by the corresponding Rrs1 receptor limits colonisation and asexual sporulation by 21 dpi) [11]. Oilseed rape resistance against *Pyrenopeziza brassicae* operates between 13 and 36 dpi [8]; thereafter leaves senesce, fungal biomass increases in resistant hosts, and sexual sporulation occurs.

e PAMPs are conserved molecules essential for microbial survival; they are constitutively produced. Effectors are race-specific proteins or peptides that suppress PTI or manipulate the host in other ways; effectors are typically induced during colonisation of the host; intracellular haustoria or BIC-forming pathogen effectors trigger ETI, as originally defined [3]. Apoplastic pathogen effectors trigger ETD. Some haustoria-forming pathogens also have apoplastic effectors to protect their hyphae [9,10].

f PRRs include chitin receptors containing extracellular LysM domains with or without a cytosolic kinase domain; other PRRs contain eLRR domains with or without cytosolic kinase domains [78]. NLRs are encoded by resistance (*R*) genes and have an intracellular localisation. RLPs are encoded by *R* genes and contain an extracellular LRR domain, a transmembrane region, and a cytosolic tail. They associate with RL kinases for signal transduction [48].

g PRRs often contain a cytosolic kinase domain for signalling. Alternatively, a PRR can interact with a RL kinase to initiate signalling [78]. N-terminal Toll/interleukin-1 receptor (TIR) and coiled-coil (CC) domains of NLRs trigger the hypersensitive response (HR) and immune response genes [58]. RLPs do not contain a known effector domain but only a short cytoplasmic tail; they require interaction with an RL kinase for signalling. The action of the RLP Cf-4 depends on the downstream target NRC1, which encodes a CC-NLR [12].

h PRRs and RLPs heterodimerise, NLRs homodimerise.

i It was recently proposed that the signalling complexes for ETD and PTI are different but BAK1 is also required for RLP function [49].

j PTI typically does not trigger plant cell death; ETI usually triggers a fast HR cell death [3]; ETD sometimes causes a delayed cell death (CD) [8].

## Box 1 R gene-mediated host resistance responses

*R* gene-mediated host resistance responses differ between those against pathogens that form haustoria or biotrophic interfacial complexes (BICs) and those against apoplastic fungal leaf pathogens (Figure I). Both types of pathogen encounter both constitutive and induced defence responses. Some of the induced defence responses are triggered by invariant PAMPs that are recognised by PRRs located on the cell surface, resulting in PTI. Both types of pathogen secrete effectors to suppress PTI. Haustoria-forming pathogens generally deliver their effectors into the host cell. Apoplastic fungal pathogens deliver effectors into the apoplast to target extracellular or membrane proteins. Intracellular effectors are directly or indirectly recognised by intracellular nucleotide binding site (NBS) leucine-rich repeat (LRR) receptors (NLRs), whereas apoplastic effectors are recognised by cell-surface RLPs [12,48]. Although intracellular recognition triggers ETI that generally eliminates the pathogen [3], extracellular recognition activates an ETD response that often involves cell wall-related defences but does not eliminate the pathogen. Molecular and phenotypic differences between PTI, ETI, and ETD are listed in Table I.
